# Supplementary material for: Immunopeptidome Diversity in Chronic Lymphocytic Leukemia Identifies Patients with Favorable Disease Outcome
Source: Cancers (Basel). 2022 Sep 25;14(19):4659. doi: 10.3390/cancers14194659 (PMC9563800; doi:10.3390/cancers14194659)
Supplement: Supplementary file 1 [file cancers-14-04659-s001.zip › cancers-1881737-supplementary.pdf]

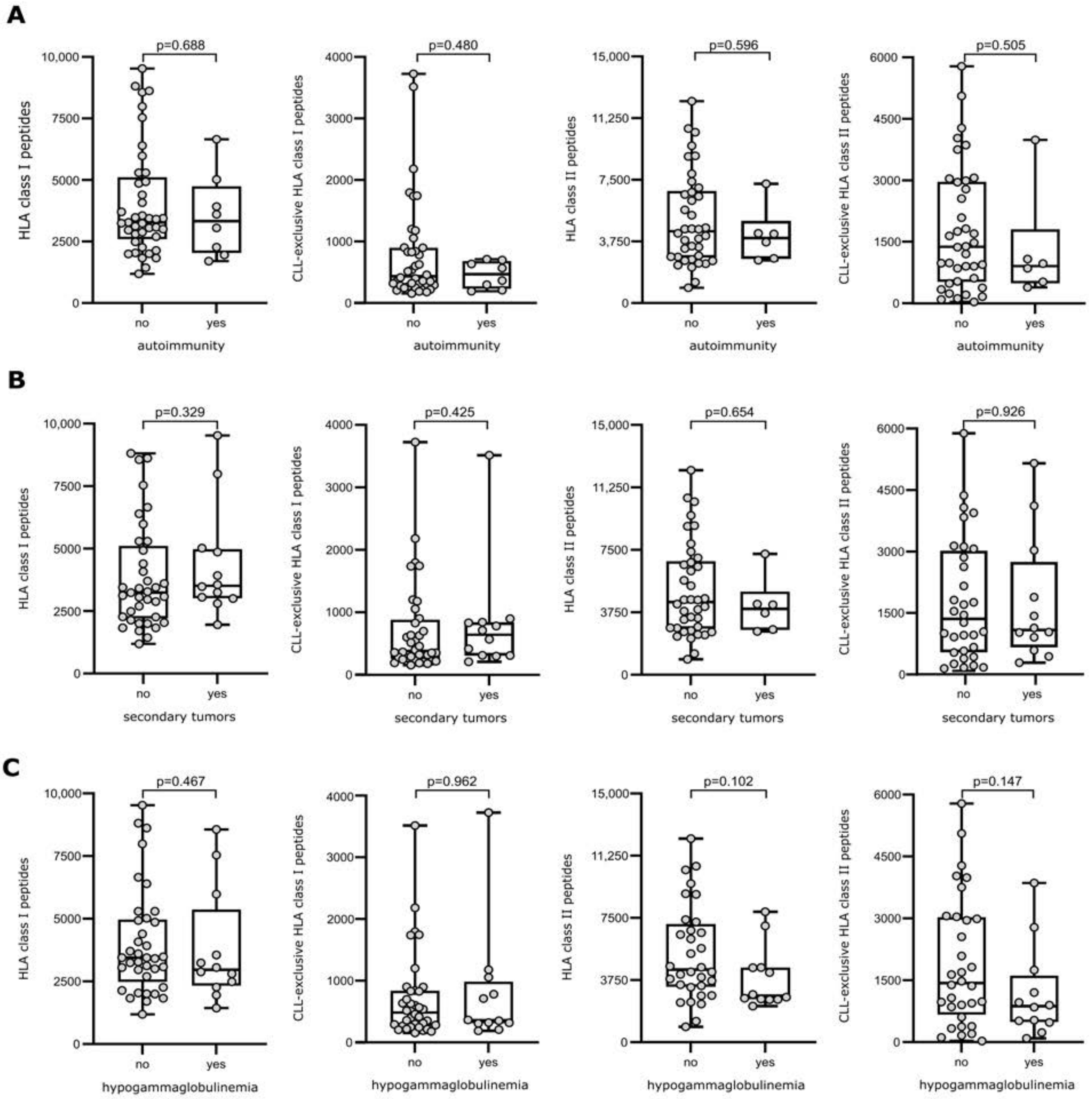

Figure S1. HLA presentation of antigenic peptides according to patients' clinical features. Number of total HLA class I- (left panel), CLL-exclusive HLA class I- (mid-left panel), total HLA class II- (mid-right panel), and CLL-exclusive HLA class II-restricted (right panel) peptides according to autoimmune phenomena (**A**), secondary tumors (**B**) and hypogammaglobulinemia (**C**). Dots represent data from individual patients. Boxes represent median and 25th to 75th percentiles, whiskers are minimum to maximum. Continuous lines indicate p-values (Mann-Whitney U test). Abbreviation: p, p-value.

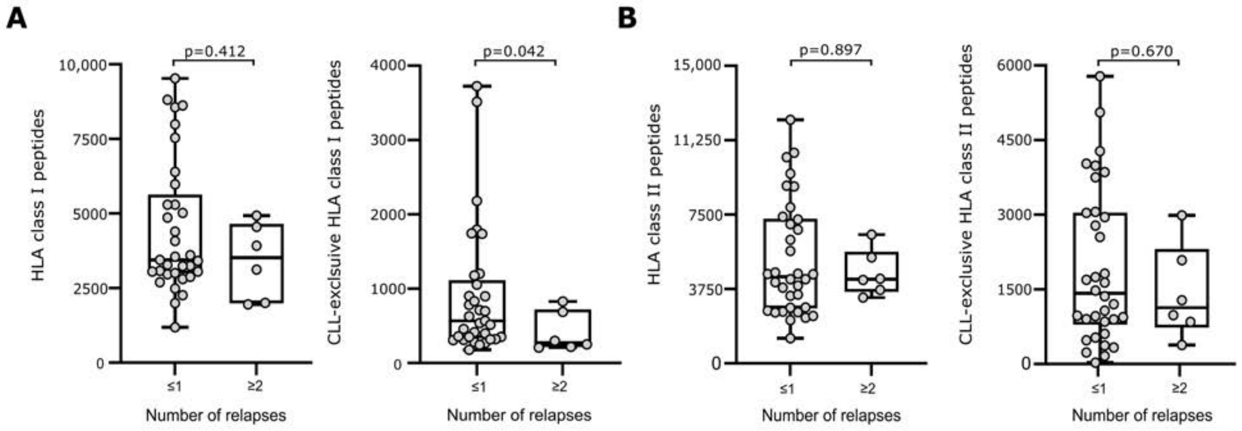

Figure S2. HLA presentation of antigenic peptides according to disease's course in terms of observed relapses analyzed in therapy-naïve patients. Numbers of total (left panel) and CLL-exclusive (right panel) HLA class I- (**A**) and HLA class II-restricted (**B**) peptides according to the number of observed relapses. Dots represent data from individual patients. Boxes represent median and 25th to 75th percentiles. Brackets indicate p-values (Mann-Whitney U-test). Abbreviation: p, p-value.

Table S1: Calculated cut-off for the definition of “high” or “low” groups for survival analysis

		Cut off values between low and high peptides' number	
		**Low**	**High**
**PFS**	HLA class I peptides	≤ 2265	> 2265
	CLL-exclusive HLA class I peptides	≤ 366	> 366
	HLA class II peptides	≤ 4504	> 4504
	CLL exclusive HLA class II peptides	≤ 1282	> 1282
**OS**	HLA class I peptides	≤ 5291	> 5291
	CLL exclusive HLA class I peptides	≤ 898	> 898
	HLA class II peptides	≤ 4245	> 4245
	CLL exclusive HLA class II peptides	≤ 1476	> 1476

Abbreviations: PFS, progression-free survival; OS, overall survival.

Table S2. Detailed patient characteristics

Patient ID	Age	Sex	Binet	IgHV	Tp53/del17	Number of HLA class I peptides	Number of CLL-exclusive HLA class I peptides	Number of HLA class II peptides	Number of CLL-exclusive HLA class II peptides
**PID01**	51	Male	B	Mutated	Negative	849	268	2801	332
**PID02**	60	Female	C	Unmutated	Positive	1308	350	Unknown	Unknown
**PID03**	53	Male	A	Unmutated	Positive	1349	782	2184	530
**PID04**	63	Male	B	Unknown	Unknown	1230	841	Unknown	Unknown
**PID05**	64	Female	A	Unknown	Unknown	1157	399	Unknown	Unknown
**PID06**	50	Female	A	Unknown	Unknown	Unknown	Unknown	2312	160
**PID07**	76	Male	C	Unknown	Negative	Unknown	Unknown	3410	941
**PID08**	49	Male	A	Unknown	Positive	728	209	4205	847
**PID09**	43	Male	A	Unknown	Positive	1121	538	Unknown	Unknown
**PID10**	57	Male	A	Unknown	Unknown	852	366	2723	518
**PID11**	59	Male	A	Unmutated	Positive	632	193	Unknown	Unknown
**PID12**	70	Female	A	Unknown	Unknown	1860	312	12,282	2954
**PID13**	75	Male	A	Unknown	Negative	679	214	Unknown	Unknown
**PID14**	71	Male	C	Unknown	Positive	Unknown	Unknown	935	201
**PID15**	72	Female	B	Unknown	Unknown	1407	600	Unknown	Unknown
**PID16**	46	Male	A	Unknown	Unknown	1243	458	2398	608
**PID17**	38	Male	A	Unknown	Unknown	2457	900	2814	28
**PID18**	64	Male	A	Unknown	Negative	1492	299	3703	382
**PID19**	57	Male	A	Unknown	Positive	759	155	6612	1386
**PID20**	67	Male	B	Unknown	Negative	1921	569	4242	1073
**PID21**	51	Female	A	Unknown	Negative	Unknown	Unknown	3814	972
**PID22**	68	Female	B	Unmutated	Positive	1182	250	Unknown	Unknown
**PID23**	90	Male	A	Unknown	Negative	1231	831	Unknown	Unknown
**PID24**	64	Male	A	Unknown	Unknown	1060	309	2661	234
**PID25**	66	Male	A	Unknown	Negative	678	291	2415	114
**PID26**	65	Male	B	Unknown	Negative	800	206	Unknown	Unknown
**PID27**	57	Female	B	Mutated	Negative	1228	322	Unknown	Unknown
**PID28**	77	Female	A	Unmutated	Negative	527	188	2884	90
**PID29**	60	Male	A	Unknown	Unknown	1554	628	3921	900
**PID30**	52	Male	B	Mutated	Unknown	3073	898	10,616	4028
**PID31**	59	Male	A	Mutated	Negative	Unknown	Unknown	4505	1201
**PID32**	53	Male	A	Unknown	Unknown	2555	636	Unknown	Unknown
**PID33**	52	Female	B	Unmutated	Negative	8561	3726	7865	3858
**PID34**	66	Male	A	Unmutated	Negative	3082	515	4096	1639
**PID35**	62	Male	C	Unknown	Negative	5976	1054	2586	477
**PID36**	60	Male	B	Unknown	Negative	8622	2181	7397	3037
**PID37**	81	Male	C	Mutated	Negative	4928	829	6488	2989
**PID38**	57	Male	B	Mutated	Negative	5298	1795	6748	3057
**PID39**	83	Male	C	Mutated	Negative	9530	3514	9570	5058
**PID40**	52	Male	A	Unmutated	Negative	3055	713	2603	962
**PID41**	66	Male	A	Unmutated	Negative	2003	219	3316	981
**PID42**	69	Male	A	Mutated	Negative	3412	282	3470	851
**PID43**	53	Male	B	Unmutated	Negative	1992	245	8922	3750
**PID44**	63	Female	A	Unmutated	Negative	4393	1200	8954	4,276
**PID45**	60	Male	A	Unmutated	Negative	8816	1738	10,392	5780
**PID46**	70	Female	A	Mutated	Negative	7539	1178	7016	2782
**PID47**	71	Male	B	Unmutated	Negative	4558	688	4272	1282
**PID48**	39	Male	A	Unmutated	Negative	2696	357	4524	1693
**PID49**	60	Female	A	Mutated	Negative	3608	699	7258	3986
**PID50**	58	Male	B	Mutated	Negative	Unknown	Unknown	4584	1476
**PID51**	77	Female	C	Unmutated	Positive	Unknown	Unknown	5343	2086
**PID52**	65	Female	A	Unmutated	Positive	Unknown	Unknown	6233	1816
**PID53**	Unknown	Female	B	Unmutated	Negative	1186	178	1264	372
**PID54**	Unknown	Male	B	Unknown	Negative	2490	319	2569	899
**PID55**	Unknown	Male	C	Mutated	Negative	3002	415	4246	1364
**PID56**	Unknown	Female	B	Mutated	Negative	2877	350	4490	1749
**PID57**	Unknown	Male	A	Unmutated	Negative	5292	1746	5673	2553

Abbreviation: PID, patient ID
